# A resilient public health in 2030: #buthow?

**DOI:** 10.1007/s12508-022-00375-6

**Published:** 2023-01-09

**Authors:** Luc Hagenaars, Wilma Waterlander, Karen den Hertog, Karien Stronks

**Affiliations:** 1 Department of Public and Occupational Health, Amsterdam Public Health research institute, Amsterdam UMC locatie AMC, Amsterdam, The Netherlands; 2 Afdeling Gezond Leven, Gemeente Amsterdam, GGD Amsterdam-Amstelland, Amsterdam, The Netherlands

**Keywords:** Leverage points, Complex adaptive systems, Systems science, Policy, Politics

## Abstract

Game changers in public health are traditionally seen as specific in(ter)ventions, but contemporary public health challenges warrant acknowledging complexity instead. Our game changer is a compass to deal with this complexity. In our vision of 2030, system beliefs, goals, structures and events line up to create a society that balances health, climate, social cohesion and economy. To reach this desired system, a resilient public health sector actively interacts with public discourse, political windows of opportunity are seized for institutionalizing health for all policies, and research is intertwined with the policy process, without the merging of the two.

## Winner of the TSG Challenge

In 2022, TSG – the Dutch Journal of Health Sciences – celebrated its 100th anniversary. To mark the occasion, TSG organised a writing contest: the TSG Challenge. We asked people in the field to write a substantiated opinion piece about the game changer for public health in 2030. The jury selected one winner from the entries and two pieces were recognised with an honourable mention.

This article is the winning piece. In its report, the jury said this about this contribution: ‘The jury beliefs that the policy concept of complexity does justice to the coherence of public health issues and is scientifically well-founded. The broad connection between health, economy, sustainability and other current major issues is adequately addressed. The jury also praises the focus on the role of professionals in the change. The approach takes a big leap forward and – by doing so – could potentially have a major impact, but it is somewhat abstract and in places too black-and-white: the desired change in 2022 is presented as a rosy picture and its description is not very concrete yet. This certainly requires further elaboration.’

## Public health in 2030

It is January 2030. Over the past years tremendous progress has been made in finding structural solutions for the major contemporary population health challenges. Acknowledging complexity emerged as the game changer. No longer are health and prevention conceptualized in terms of determinants, individual behaviour, and lifestyle interventions. Instead, we have come to regard population health as the outcome of a system in which a wide variety of elements related to social cohesion, climate, and economic prosperity interact [1].

Moreover, we now have a balance between health, social cohesion, climate, and economy. In the beginning of the 21st century, economic growth was still the single most important societal goal. It was practically impossible to pursue goals that did not directly or indirectly contribute to growth, let alone pursue goals that reduced growth. An example. In 2022, 80% of our food environment was unhealthy. Junk food was more profitable than healthy food. As a result, a small number of companies made enormous profits, and, consequently, their large marketing budgets and political lobbies maintained the status quo. Until society rebelled against this system. When the public became protected against the overabundance of cheap highly-palatable, ultra-processed food, unhealthy and environmentally harmful food became less profitable. But that was not all. In 2030, the following health protective measures have become so normal, that we hardly notice their existence: a speed limit of 80 km/hour on ring roads, sustainability and liveability form the basis for local policies, and secured livelihood is the guiding principle in social security systems.

The future outlined here might have seemed utopian in 2022, but it was not. After all, complex adaptive systems such as population health—in which interconnected components are continuously adapting to changes from within and from outside the system—are, by definition, dynamic. For example, we were able to abolish slavery, something that was once deemed impossible. Moving towards a new system does not require superhuman efforts, but primarily calls for thinking and acting from the perspective of complex systems [2].

The shift in thinking about population health from a complex systems perspective started halfway the 2010s. This cautious start did not immediately translate to practice in the Netherlands, as evidenced by the first Prevention Agreement’s focus on individual lifestyle choices in 2018, and its consequential lack of health impact. Nevertheless, in part because the COVID-19 pandemic clearly showcased structural health inequalities, health was increasingly seen as a product of society as a whole [3].

The compass for dealing with complexities lies in the four interacting dimensions of systems change which Meadows [2] first identified. In order of importance these concern: 1) the specific policy measures as described above; 2) the structure within which these measures are implemented; 3) their policy goals; and 4) underlying belief systems. We describe how focusing on beliefs, policy goals and the structure led to a resilient public health in 2030. Resilient meaning a public health that is based on a thorough comprehension of the workings of the complex system that produces population health, and that acts strategically in line with the system changes required to improve population health. Tab. 1 summarizes the leading principles before and after this transition.

**Table 1:** Central principles of public health in 2022 and 2030. Dimensions based on Meadows [2]

2022	2030
*Dimension 1—convictions*	
Lifestyle	Environment
Determinants	Underlying mechanisms
Health interventions	Making changes to the system that produces (ill) health
Health promotion	Health protection
Individual responsibility	Collective responsibility
Healthy choice easy choice	Healthy environment
Selective prevention	Proportional universalism, i.e., the resourcing and delivery of universal services at a scale and intensity proportionate to the degree of need
Economic interests and public health care are conflicting	Population health is a predisposition for a healthy economy and vice versa
*Dimension 2—policy goals*	
Intersectoral policy	Policy goals integration
Health* in* all policies	Health* for *all policies
Searching for possibilities within the existing policy goals	Health goals interwoven with the goals of relevant sectors
*Dimension 3—structure*	
One problem owner	Multiple problem owners
Health monitor	Broad welfare monitor
Translating scientific knowledge into policy	Researchers and policymakers are partners in policy development
Research methods to find out what works (attribution)	Research methods to understand what contributes to (contribution)
Advise other sectors how they can improve health	Help other sectors with their own goals as well as their health-related goals
*Dimension 4—measures*	
Lifestyle interventions (for example combined lifestyle interventions or cooking classes)	Leverage points in the physical, social, economic, and digital environments (for example, walkability of facilities and services, social norms regarding sunbathing, healthy food security, and a ban on cryptos and gambling)

## Dimension 1—Beliefs in 2030

Which beliefs proved crucial in sustaining systemic changes? We will name two: how we conceptualise health and how we weigh health against other societal goals.

### Conceptualisation of health

Whereas health had long been perceived as dependent on chance (genetics) and individual behaviour, the COVID-19 pandemic showed us that this was factually incorrect. In 2022, the Health Council of the Netherlands (*Gezondheidsraad*) wrote “the burden of disease in humans is predominantly determined by environmental factors” [4]. The way that policy makers and politicians think has changed. Referring to the social and environmental context of behaviours is no longer the exception, but has become part of the regular public and political debate, because lay people and politicians now perceive health as a product of society instead of as an individual’s responsibility.

The COVID-19 pandemic highlighted this reversal in thinking, and consequently more people got involved in the ongoing discourse about equal opportunities. Particularly successful (and generally influential) people had long assumed that their success was the result of their individual competencies. However, the increasing discrepancy in opportunities confronted them with a reality that those who were less successful had known all too well, namely that people on a lower socioeconomic position did not choose to be unhealthy. The realisation that health is not a choice really gained momentum when, because of climate change, extreme weather conditions more and more frequently posed health risks in the Netherlands as well.

### Health in relation to other societal goals

The changes in beliefs described above did not come easy. The public health sector was forced to self-reflect when it initially failed to capitalize on the window of opportunity for prevention that the COVID-19 pandemic created. Evaluation showed that public health could have been better attuned to the beliefs of the political majority. A political majority denounced the idea that the pandemic was a threat to the economy. That is why in the late 2020s, public health experts positioned health as a condition for economic growth. Instead of public health measures being seen as a threat to the economy, there now is growing awareness that certain industries are detrimental to population health, and therefore also for economic growth. It became clear that the *devil shift*, in which bad qualities, bad goals and bad behaviour of opponents in policy disputes are exaggerated [5], helped to force a breakthrough. This tactic has previously been deployed successfully against the tobacco industry, and in the year 2030 this success is repeated; this time with the food and gaming industries taking the deservedly blame.

## Dimension 2—Policy goals in 2030

How did the policy goals change in line with the shifts in beliefs? The Punctuated Equilibrium Theory proved to be a good basis for institutionalizing health in the goals of policy systems [5]. According to this theory, politicians do not have the ability to adequately consider all problems at the same time. Policy silos claim ownership of a theme and have an interest in maintaining the status quo. Intersectoral collaboration will only succeed if ownership of the problem is not under threat. However, when the societal perception of a problem tilts, another dynamic comes into play. Political leaders then will (temporarily) become active problem owners, with the aim to fundamentally change policy.

### Legally mandated public health benchmarks in 2030

In the 2020s, health professionals seized these tilting beliefs with both hands to argue in favour of legally mandated public health benchmarks [6]. These benchmarks institutionalised permanent focus on health compared to other goals, similar to climate benchmarks. It took a while to come up with a health equivalent for carbon dioxide emissions, formulated in such a way that politicians could be held responsible. Epidemiological efforts to measure underlying, controllable environmental health hazards, such as fast-food outlet density in areas and an extensive taxonomy of lack of secured livelihood, nonetheless provided a valid set of measuring tools.

These “legally mandated public health benchmarks” had major effects. Because the objectives were well aligned with the climate goals and general welfare goals that go beyond GDP, health became part of a policy integration instead of a whole of government approach. The difference can be explained as follows. Whole of government implies intersectoral collaboration between various public service delivery actors, such as healthcare, education or social services, and mainly involves (re)distribution of means as action instrument. The public health sector has a coordinating role in that process when it comes to health. In view of the original dominance of health promotion interventions (see Tab. 1), this often resulted in a focus on health promotion in vulnerable groups. This approach was only marginally effective, because it failed to tackle social and environmental risk factors. Policy integration, on the other hand, starts by establishing goals for the various policy silos, after which the responsible sectors manage implementation within their respective silos themselves, with regulation as the primary action instrument [7]. Policy-making officials who shape the environment (such as spatial planning, sustainability, economic affairs departments), proved to be very good at this. When spatial planning became problem owner of the food environment, for example, policies for reducing the supply of unhealthy food were quickly implemented.

### Better fragmented policy

Public health officials created a second breakthrough by systematically supporting non-health policymakers in achieving their health, and non-health goals. An example. Many children attend school without having eaten breakfast. When it emerged that this was one of the contributing factors to the deterioration of reading and writing skills, a school breakfast programme was gradually introduced into schools where many pupils arrive hungry.

With this constructive position, the public health sector had finally abandoned the one-way tradition of forcing public health interests unto other sectors. Health *in* all policies has been replaced by the term health *for* all policies [8], and it does not stop with semantics. The public health sector has gotten skin in the game: it is, for example, rapidly setting up appropriate research to help tackle the upcoming challenges in relevant sectors. In the year 2030, public health policy integrated well with other sectors, and this is coming from both ways.

## Dimension 3—The public health structure in 2030

The change of policy goals came with structural changes. We focus on two aspects: how research and policy interact, and the competences of professionals.

The Netherlands have always had a strong structure for health monitoring. This structure was supplemented with data on underlying social and environmental determinants after the COVID-19 pandemic highlighted their importance, and because it tied in with the already ongoing development to put broad welfare centre stage instead of the GDP [9].

Monitoring is not an isolated process but is interwoven with policy development. In a joint effort, teams of researchers, policy makers, social organisations and civilians analyse the complex of underlying causes of an emerging public health issue, how that system can be changed, and what the consequences of such a change will be for other societal goals. On the one hand, there is the recognition that knowledge is not policy neutral, and on the other hand the recognition that policy needs knowledge. This recognition was crucial to evidence-informed policy.

These structural changes could only take place because the competences of public health professionals became aligned to the desired future. Research shows that the ultimate game changer of public health, sewage disposal, was indebted to the political sensitivity, the research capacities, and the efforts of hygienists to help the less fortunate [10]. The latter two competences only needed to be calibrated to the complexity of current public health issues. The political competences of public health professionals required more revitalisation efforts.

## In a flash, breaking news throws you back to the realities of December 2022

The new variant of the coronavirus is a more significant reason for concern than anticipated; again a lockdown seems necessary. While you are digesting this news, you and your policy studies team are putting the finishing touches to the implementation plan of one of the legally mandated public health benchmarks. As you are searching for words that can help change current beliefs of health as a matter of luck and individual responsibility, you wonder whether this plan will trigger the utopian feedback mechanisms as described above to produce a resilient public health …
